# A Multidirectional Non-Cell Autonomous Control and a Genetic Interaction Restricting Tobacco Etch Virus Susceptibility in Arabidopsis

**DOI:** 10.1371/journal.pone.0000985

**Published:** 2007-10-03

**Authors:** Suresh Gopalan

**Affiliations:** Institute of Biological Chemistry, Washington State University, Pullman, Washington, United States of America; Massachusetts General Hospital and Harvard Medical School, United States of America

## Abstract

**Background:**

Viruses constitute a major class of pathogens that infect a variety of hosts. Understanding the intricacies of signaling during host-virus interactions should aid in designing disease prevention strategies and in understanding mechanistic aspects of host and pathogen signaling machinery.

**Methodology/Principal Findings:**

An Arabidopsis mutant, B149, impaired in susceptibility to Tobacco etch viru*s* (TEV), a positive strand RNA virus of picoRNA family, was identified using a high-throughput genetic screen and a counterselection scheme. The defects include initiation of infection foci, rate of cell-to-cell movement and long distance movement.

**Conclusions/Significance:**

The defect in infectivity is conferred by a recessive locus. Molecular genetic analysis and complementation analysis with three alleles of a previously published mutant *lsp1* (loss of susceptibility to potyviruses) indicate a genetic interaction conferring haploinsufficiency between the B149 locus and certain alleles of *lsp1* resulting in impaired host susceptibility. The pattern of restriction of TEV foci on leaves at or near the boundaries of certain cell types and leaf boundaries suggest dysregulation of a multidirectional non-cell autonomous regulatory mechanism. Understanding the nature of this multidirectional signal and the molecular genetic mechanism conferring it should potentially reveal a novel arsenal in the cellular machinery.

## Introduction

Plants, humans and other hosts are constantly challenged by a wide variety of pathogens in the environment, including viruses, fungi, bacteria, insects and nematodes. While hosts have evolved a variety of mechanisms to recognize and counter pathogens, the rare combinations leading to disease cause significant damage to the host. Innate immunity in plants and animals involve many analogous signaling modules but it is not known whether they evolved as a consequence of convergent or divergent evolution [1]. Viruses as a class of pathogens are typically countered by many of these conserved host mechanisms. Many viral components are recognized in a manner similar to other pathogens, for example using pattern recognition receptors (PRRs) including Toll like receptors (TLRs) in mammals and other LRR-containing intracellular receptors [2], [3] such as R proteins that recognize viral components in plants that in some cases elicit cell death inducing pathways [4]–[6]. In addition, a major component of defense against many classes of viruses in a variety of hosts have been shown to involve components and pathways involved in RNA silencing (e.g., [7]–[14]). Results from a forward genetic study to uncover additional host components and mechanisms during infection by a plant positive strand virus are presented here.

*Tobacco etch virus* (TEV) is a positive strand RNA virus, that broadly belongs to the picoRNA virus family, and to the potyvirus family in plants. The genome of TEV is translated as a single polypeptide which is processed by a series of *cis* and *trans* acting proteolytic clevages that are encoded by the viral genome, thus generating the functional viral proteins [15], [16]. Some of the proteins include the viral RNA dependent RNA Polymerase (RdRP) NIb, CI (cylindrical inclusion protein) that has helicase activity, at least three proteins with proteolytic activity NIa, and P1 and HC-Pro, and the capsid protein. TEV, like other plant viruses moves cell-to-cell through plasmodesmata. In susceptible plants, TEV moves systemically with the assimilate transport through the phloem. At least four of the TEV encoded proteins (capsid, CI, NIa and HC-Pro) have been shown to facilitate virus movement, capsid and CI in cell-to-cell movement and all four proteins in long distance movement [17]. TEV tolerates insertion of foreign genes, thus permitting construction of reporter and selectable viruses. Recombinant TEV with the reporter genes GUS and GFP [18] has been extensively used to study the roles of the viral encoded proteins.

TEV infects *Arabidopsis thaliana* plants in an ecotype specific manner. Infection of a variety of Arabidopsis ecotypes with TEV leads to no visible phenotype in the plant. However, TEV-GUS inoculated on leaves of C24, a susceptible ecotype, forms foci that can be visualized by GUS staining not only on inoculated leaves but also on uninoculated floral tissue by 10 days post inoculation (dpi). In contrast, in the restrictive ecotype Col there is no systemic movement, although foci formation on inoculated leaves is similar to C24 [19]. One well studied form of disease resistance in plants involves the recognition of pathogen factors by plant resistance (R) genes often accompanied by a rapid plant cell death at the site of infection, termed the hypersensitive response (HR)–[20], [21]. The restriction of TEV in Col ecotype is not mediated by HR type resistance, as evidenced by lack of macroscopic cell death lesions at sites of infection. Further, mutants impaired in HR type resistance, and plants in which salicylic acid (a key signaling molecule in HR mediated defense) is converted to catechol by a *nahG* transgene, do not have any effect on the restriction of systemic movement in Columbia ectoype plants [19]. In contrast, RNA interference and post transcriptional gene silencing (PTGS) seems to be a major contributor of resistance to this class of viruses (some early examples include refs. [22]–[27]). Genetic analysis of the ecotype specific difference in infectivity and mutagenic analysis have revealed three components involved in the restriction of TEV movement in Col plants, RTM1, RTM2 and RTM3 (RTM–restriction of TEV movement)-([19], [28], [29]; unpublished results).

Arabidopsis mutants corresponding to components not directly involved in HR-type resistance were identified in studies utilizing immunological screens for the levels of viral coat proteins. Mutants *tom1* and *tom2* exhibit reduced susceptibility to *Tobacco mosaic virus* (TMV) [30], [31] and mutants *cum1* and *cum2* [32], [33] are less susceptible to *Cucumber mosaic virus* (CMV). While *tom1* and *tom2* were affected in multiplication of TMV, *cum1* was affected in local spreading of CMV in inoculated leaves, and *cum2* was affected in cell-to-cell movement of CMV and TCV. Two Arabidopsis mutants *vid1* and *vsm1* were identified by loss of viral infection phenotypes for TMV and TVCV, respectively [34], [35]. *vsm1* restricts TMV to inoculated leaves, while *vid1* develops a dwarf phenotype upon infection with *Turnip vein clearing virus* (TVCV). Several other loci that are involved in HR type resistance to viruses have also been identified in Arabidopsis and other plants. Examples of well studied *R* gene mediated resistance to viruses include *N* gene mediated resistance to TMV and Rx gene mediated resistance to Potato virus X (PVX)–[4], [5], [36], [37].

With the aim of identifying additional components involved in virus-host interactions a genetic screen to identify mutants affected in TEV infectivity in the susceptible ecotype C24 was undertaken.

## Results

### Isolation of altered susceptibility mutants using TEV-P450 selection

Inoculation of the susceptible Arabidopsis ecotype C24 with TEV results in formation of infection foci in inoculated leaves and long distance movement of the virus to uninoculated parts of the plants (typically tested in the floral tissue). But this infection does not result in any visible phenotype. Thus the counterselectable virus TEV-P450 developed earlier was used to screen for Arabidopsis mutants impaired in susceptibility to TEV. TEV-P450 has the gene coding for P450-SU1 (from *Streptomyces griseolus*) inserted into the TEV genome with a chloroplast targeting signal [38], [39]. The P450-SU1 product when expressed in plants converts the proherbicide R7402 to a toxic herbicide.

EMS mutagenized C24 plants (M2 generation) were inoculated with TEV-P450, and sprayed with R7402 in a spray chamber 10 and 12 dpi. Most plants were dead or very necrotic due to the conversion of R7402 to the toxic herbicide form. The differential between plants affected by the conversion of the proherbicide R7402 to the toxic herbicide form and the unaffected plants was clear within a week. In total 33,000 mutagenized seeds were screened and 62 putative mutants identified. The putative mutants (plants that had less symptoms or were asymptomatic) were analyzed for infectivity and systemic movement with TEV-GUS in the M3 generation in comparison to parental C24 ecotype. This step eliminated plants that were escapes in one of the steps of screening and mutants in the herbicide response pathway. The characterization of one mutant-B149, that was affected in its susceptibility to TEV, is described below.

### Phenotypic characterization of B149 reveals specific loss of susceptibility to TEV by a non-cell autonomous mechanism

**Figure 1:**
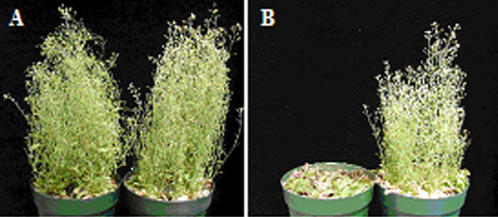
Response of B149 and C24 plants to TEV-P450/R7402 counterselection. B149 (Panel A) or C24 (Panel B) plants were mock inoculated (pot on right) or inoculated with TEV-P450 (pot on left). Plants were sprayed with R7402 10 dpi and 12 dpi. Symptoms in this figure was recorded 24 days later. The effect of R7402 on wild type C24 infected with TEV-P450 can be distinguished within the first few days of R7402 application.

As expected B149 plants were resistant to infection with TEV-P450 followed by R7402 treatment, whereas C24 plants were almost dead (Figure 1). B149 plants were also modestly smaller than wild-type and were slightly chlorotic. (Footnote of Table 1). However, when B149 was backcrossed to wild-type C24 plants, the small size and chlorotic traits could be segregated away from the inhibition of foci formation phenotype in leaves (data not shown).

**Table 1:** Development of infection foci on C24 and B149.

Foci/Plant	C24*	B149*	p value
E1	93.4 (5)	4.9 (10)	2.4 e-6
E2	343.4 (5)	12.05 (18)	9 e-11

Plants inoculated with TEV-GUS were infiltrated with X-gluc four dpi (E1) or three dpi (E2), and the number of visible blue foci were counted.

* Average of number of foci (number of plants).

*Average plant weight (29 day old plants) during experiment 2 (B149: 0.67; C24:1.17. p = 3.8e-6).*

**Figure 2:**
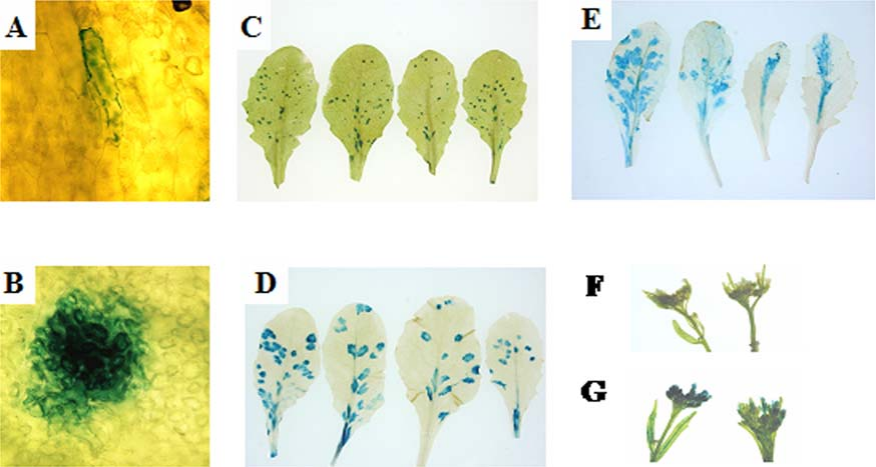
Dynamics of TEV-GUS infection in C24. Arabidopsis plants (C24 ecotype) were mock inoculated or inoculated with TEV-GUS. Leaves or inflorescence tissue were infiltrated with X-gluc at the time points indicated. Pictures at 1 dpi (Panel A) and 2 dpi (Panel B) are photomicrographs at 200× magnification (with 20× objective and WH10 eyepiece). Panel C is leaves from 3 dpi. In case of 8 dpi (Panel D) and 16 dpi (Panel E), leaves were bleached following color development. Panels F and G are floral clusters from mock inoculated plant and TEV-GUS inoculated plant, respectively (18 dpi).

As previous work in tobacco and Arabidopsis has detailed, infection of TEV typically starts in a single cell and spreads to adjacent cells through plasmodesmata to form visible foci (when stained for GUS activity) in the first two to three days. The foci continue to spread on inoculated leaves and to other parts of the plant. By 10 dpi GUS activity can be detected in the floral tissue, indicative of systemic movement of TEV-GUS. B149 was characterized for susceptibility to TEV-GUS at various stages of infection. The different stages of infection and progression in Arabidopsis is shown in Figure 2 in order to better understand the analyses of each of these steps presented below.

**Figure 3:**
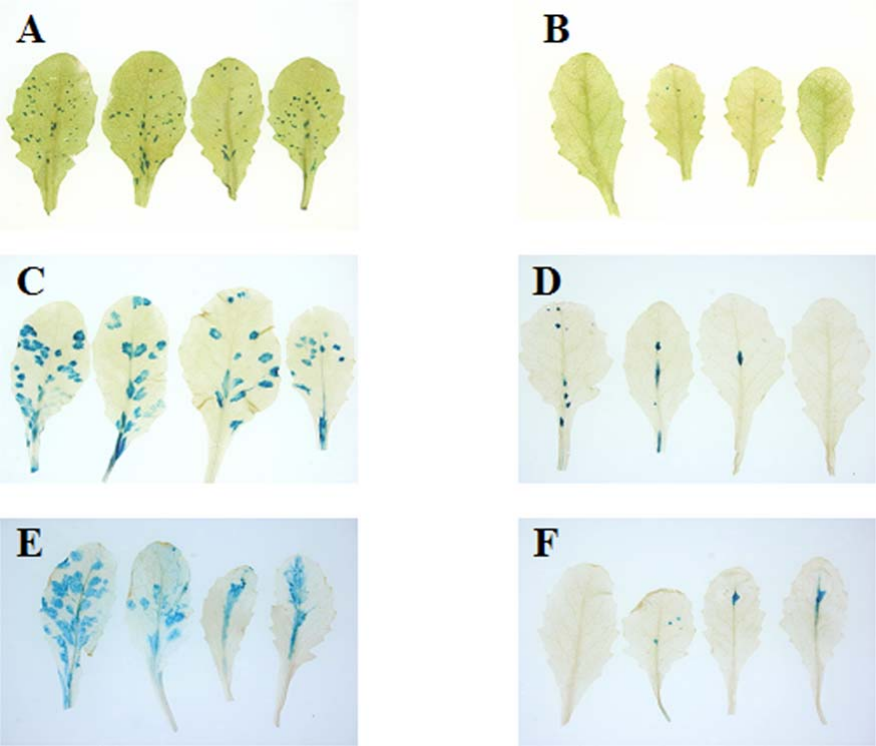
Foci development of TEV-GUS in infected leaves of C24 and B149. Leaves from C24 (Panels A, C, E) or B149 (Panels B, D, F) plants inoculated with TEV-GUS were infiltrated with X-gluc after 3 dpi (Panels A and B), 8 dpi (Panels C and D), and 16 dpi (Panels E and F). The leaves from 8 dpi and 16 dpi plants were bleached post color development.

Quantitation of the number of foci formed per plant revealed that B149 had 20 fold lower visible foci in two independent experiments (Table 1). As can been seen in Figure 3, the number of foci formed on B149 were significantly lower than the number of foci on parental C24 plants inoculated in parallel. The striking nature of the visual difference between wild-type and B149-infected plants and the uniformity can be seen in supplemental Figure S1. It can be indirectly inferred that the reduction in the number of foci was not simply a consequence of the slow expansion of foci from the observation that the number of foci on B149 plants were significantly lower than on C24 even at later time points (Figure 3, Panels B and C). Thus, there is a suppression of viral foci initiation and foci expansion in the B149 mutant.

**Figure 4:**
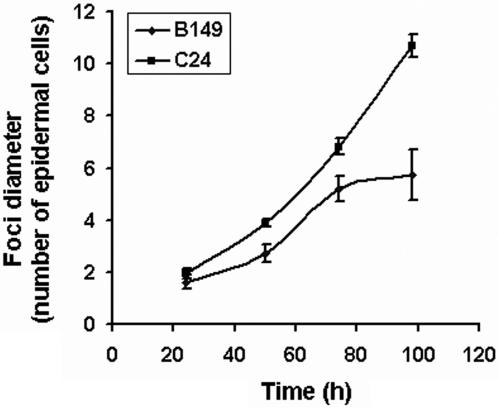
Rate of cell-cell movement of TEV-GUS in inoculated leaves of B149 and C24. Leaves from plants inoculated with TEV-GUS were harvested at time points indicated and infiltrated with the colorimetric substrate X-gluc. Foci diameter were measured microscopically at time points indicated and represented as mean (±SD). Data at 24 h represent mean of 17 foci, other points represent mean of 39 foci.

The rate of cell-to-cell movement of TEV-GUS in B149 was compared to that of C24 by measuring the diameter of infection foci microscopically (i.e., epidermal cells expressing GUS activity) at different time points. B149 had some impairment in the rate of cell-to-cell movement (foci diameter of 2.72 cells versus 3.88 cells in parental genotype) as early as day 2. While the limited foci that formed on B149 expanded slowly and stopped expanding after a few days, foci on C24 continued to expand rapidly (Figure 4 and Figure 3-Panels B and C). These two phenotypes i.e., reduction in foci initiation and expansion will be referred to as SLIM, for suppressed leaf infection and cell-to-cell movement.

There were two exceptions to the SLIM phenotype, (i) when a foci was on or in contact with the midrib it seemed to expand rapidly, (ii) also the restriction was not as succinct when the foci were at the edge of the leaf (elaborated later). These data suggest a non-cell autonomous control mechanism affecting TEV susceptibility and likely propagating towards or from the source of infection that is dysregulated in B149 (discussed further later).

**Figure 5:**
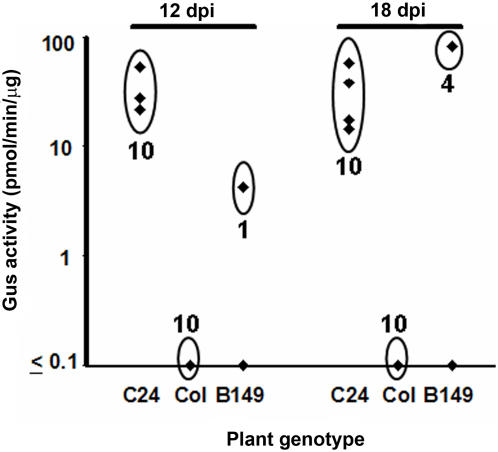
Systemic movement of TEV-GUS in C24, B149 and Col plants. GUS activity, at time point indicated. Data for both time points are from the same set of 10 plants. All plants were confirmed to be successfully inoculated by detection of infection foci in inoculated leaves by infiltration of the colorimetric substrate X-gluc. Since data points overlap, the number of independent points represented (out of ten) are indicated in the graph.

The ability of TEV to move long distance in B149 was examined by assaying GUS activity in floral tissues at the indicated time points. As can be seen in Figure 5, at 12dpi only one of ten B149 plants had GUS activity in floral tissue, whereas all ten C24 plants analyzed had higher GUS activity than the lone B149 plant that had GUS activity. Even at 18 dpi only four of 10 B149 plants had GUS activity. As expected, none of the Col plants had any GUS activity in the floral tissue due to the restriction of long distance movement in this ecotype (Figure 5).

**Figure 6:**
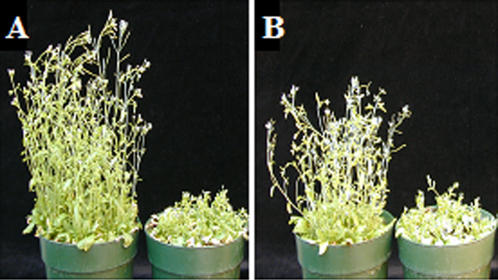
Infectivity of C24 and B149 with TuMV. C24 (Panel A) or B149 (Panel B) plants were mock inoculated (pot on left) or inoculated with TuMV (pot on right) and pictures taken 10 dpi. Leaves from *Nicotina benthamiana* plants 6 dpi with TuMV was used for inoculating plants.

Next the susceptibility of B149 to a related potyvirus *Turnip Mosaic Virus* (TuMV) was examined. TuMV has an identical genome organization and has about 50% identity (and 67% conservation) to TEV at the protein level, but Arabidopsis (in this case C24 ecotype) displays a higher susceptibility to TuMV, and causes severe stunting, leaf curling and defects in floral organ formation. Interestingly, there was no visible difference in infection phenotypes between C24 and B149 inoculated with TuMV (Figure 6). In fact, there was no difference in timing of appearance of symptoms between the two genotypes (data not shown).

B149 was also completely susceptible to the *Carmovirus Turnip crinkle virus* (TCV). There was no difference in infection phenotypes between B149 and wild type C24 plants inoculated with TCV (in timing and visual appearance of maceration symptom, which eventually cause death of plants)–(supplementary Figure S2 and data not shown). In both these cases any subtle quantitative differences remains to be explored.

### Genetic Analysis of SLIM phenotype in B149 mutant

The suppressed leaf infection and movement (SLIM) phenotype of B149 was genetically characterized. In scoring the SLIM phenotype, reduced and restricted foci on leaves was considered to be the primary phenotype whereas leaves with foci on or in contact with the midrib or at the edge of the leaves were not scored. The latter two criteria served to exclude mis-scoring the primary SLIM phenotype as these latter foci are not succinct and restricted as in the rest of the leaves, as discussed above.

F1 plants from a backcross with parental C24 plants displayed wild type leaf infection and movement, indicating that the SLIM phenotype is recessive. F2 plants analyzed for the SLIM phenotype showed a 3∶1 segregation of wild type:SLIM phenotype, indicating SLIM is likely conferred by a recessive locus (Table 2). F1 and F2 plants from crosses of B149 with ecotype Ler, generated for the purpose of mapping and analyzed for the SLIM phenotype, also displayed similar characteristics as the backcrossed plants (Table 2). In addition, a comparable number of F2 plants from crosses between C24 and the Ler ecotype that were analyzed showed that the above criteria used to score SLIM phenotype was very reliable.

**Table 2:** Genetic Analysis of B149 mutant

Genetic background	Wild type leaf movement	Restricted leaf movement
C24	10/10	0/10
B149	0/12	12/12
C1221	0/10	10/10
C1221 X B149	0/10	10/10
C13-3	5/6*	0/6
C13-3 X B149	10/10	0/10
C15-8	0/10	10/10
C15-8 X B149	0/10	10/10
C18-78	10/10	0/10
C18-78 X B149	10/10	0/10
C24 X B149	8/8	0/8
B149 X Ler/F2†	71/96	25/96
C24 X Ler/F2	75/75	0/75

Leaves from plants inoculated with TEV-GUS were collected 10 or 11 dpi, infiltrated with X-gluc and scored for status of TEV-GUS movement 24 h later, by the criteria outlined in the text. Data for crosses are from F1 plants unless indicated otherwise.

* the only plant with no foci did not have any good leaves for sampling at this stage, but had GUS activity in systemic tissue confirming infection.

† χ^2^ = 0.432 for 3∶1 segregation wild type:mutant phenotype.

TuMV has a severe stunting effect on the Arabidopsis C24 ecotype, besides causing other defects in leaf and floral organ formation (Figure 6). While the work described in this paper was in progress, independent efforts were undertaken to exploit this phenotype as a positive selection strategy to identify C24 mutants impaired in their susceptibility to TuMV, [40]. Genetic crosses were carried out with four of the mutants (belonging to two allelic groups) identified in that screen as defective in infectivity to TuMV. As indicated in Table 2, the mutation causing the SLIM phenotype in B149 is allelic to the mutations in C1221and C15-8 impaired in TuMV susceptibility, both of which belong to the same allelic group, termed *lsp1* (*lsp*-loss of susceptibility to potyviruses). However, B149 is not allelic to C13-3 that does not belong to the same complementation group as *lsp1*, and is not impaired in susceptibility to TEV (Table 2).

The mutants C1221 and C15-8 (*lsp1-1* and *lsp1-2*, respectively) impaired in TuMV susceptibility, are also impaired in a leaf infectivity phenotype to TEV-GUS as presented in Table 2 and data presented by Lellis et. al. 2002. *lsp1-1* and *lsp1-2* have been shown to harbor nonsense mutations in the gene *eiF(iso)4E* and the loss of susceptibility to TuMV in the case of *lsp1-1* can be rescued by transforming the wild type gene from C24 [40]. The *eiF(iso)4E* gene product has been shown to interact with VPg of TuMV [41]. Also a tomato *eiF4E* homolog has been shown to interact with NIa of TEV [42]. In one case, complex formation between eiF4E and the TEV encoded VPg has been shown to correlate with virus infectivity [43]. More recently, an Arabidopsis line with a transposon insertion in the *eiF(iso)4E* gene has also been shown to be resistant to TuMV and Lettuce mosaic virus (LMV)–[44]. Besides being a translation initiation factor, eif(iso)4E has also been shown to bind 5′ Cap structure of eukaryotic mRNAs [45], [46].

*eiF(iso)4E* was sequenced from the parental genotype C24 and from the mutant genotype B149. Sequence comparison revealed that B149 had a G to A transition in the last base of the third (of four) introns (splice acceptor site). The first three exons code for 186 of total 219 amino acids of the eiF(iso)4E protein. The data presented above suggest that the splice-site mutation in *eiF(iso)4E*, as opposed to nonsense mutations at codons 63 or 120 in *lsp1-1* and *lsp1-2*, respectively [40] or disruption of the gene by transposon insertion [44], might be responsible for the narrower range of impairment of B149 (only for susceptibility to TEV and not for TuMV).

### The genetic locus conferring SLIM phenotype in B149 is distinct from *lsp1* but interacts with *lsp1*

The information presented above about the B149 mutant, viz., a single recessive locus (Table 2), lack of complementation of the B149 phenotype in the F1 generation of crosses between B149 and two independent *lsp1* alleles *lsp1-1* and *lsp1-2* in terms of susceptibility to TEV, and a molecular lesion consistent with EMS mutagenesis in the gene conferring *lsp1* phenotype, points to the fact that SLIM phenotype in B149 is conferred by the same gene as the one conferring impairment of susceptibility to TEV in *lsp1*. On the other hand, in contrast to the conclusion that the B149 phenotype is a consequence of a lesion in *eiF(iso)4E*, the SLIM phenotype B149 does not appear to be allelic to C18-78 (Table 2), previously classified as an allele of *lsp1* (*lsp1-3*). C18-78 was previously reported to contain the same mutation as *lsp1-2* (in addition to an undisclosed point mutation in an upstream intron). In addition, C18-78 does not seem to be affected in susceptibility to TEV as in the case of C13-3 that belonged to a different complementation group (Table 2).

Strikingly, analysis of a small segregating population of F2 plants exhibiting the SLIM phenotype, from a cross between B149 and Ler (an ecotype that permits long distance movement of TEV), using two sslp markers from BAC clones MOK9 and MEE13 [40] (about 450 kb apart-about 120 kb south and 300 kb north of Lsp1 (eiF(iso)4E)-and spanning the locus conferring *lsp1* phenotype) indicated that there is an incomplete linkage (Table 3 and detailed in next section) of the SLIM phenotype to the same region on chromosome V as *lsp1*. This is inferred from the fact that over 25% of the very small population of backcrossed F2 plants analyzed were heterozygous at both markers, despite retaining the SLIM phenotype. These data indicate that the restriction of SLIM phenotype in B149 is not through the loss of function of eiF(iso)4E in B149, and that a synthetic genetic interaction between the B149 locus and *lsp1* locus causes a haploinsufficiency, thus displaying a non-allelic non-complementation phenotype. It should be highlighted that there were no observable differences in SLIM phenotype or systemic spread in several generations of descendents of this mutant.

**Table 3:** Molecular linkage analysis of B149 mutant

Line	MOK9–39S	MEE13-435
BL1	H	H
BL3	C24	C24
BL4	C24	C24
BL5	C24	C24
BL12	C24	C24
BL20	C24	C24
BL30	C24	C24
BL31	C24	C24
BL37	C24	C24
BL39	C24	C24
BL42	C24	C24
BL46	C24	C24
BL47	H	H
BL48	H	H
BL59	C24	C24
BL64	C24	C24
BL68	ND	C24
BL73	ND	C24
BL78	ND	Ler
BL91	ND	C24
BL92	ND	C24
BL108	C24	C24
BL112	H	H
Ler	0	1
Het	4	4
Total	19	24

Analysis was performed on F2 plants from B149 X Ler population with B149 phenotype in terms of leaf infectivity.

Leaves from plants inoculated with TEV-GUS were collected 10 or 11 dpi, infiltrated with X-gluc and scored for status of TEV-GUS movement 24 h later, by the criteria outlined in the text.

H, Het–indicates heterozygous for that marker; ND–Not determined.

### A non-cell autonomous multi-directional signal operative in B149 affecting susceptibility to TEV

As mentioned earlier, the foci formed on the leaves of B149 that were on or in contact with the midrib seem to expand more rapidly. This raised the possibility that the defect in foci formation and expansion was restricted only to some cell types present in leaves. To test this possibility, bolts of C24 and B149 plants were inoculated with TEV-GUS. All C24 plants had GUS activity in floral tissue (fluorometric GUS assay) whilst floral tissue from all the B149 plants had no GUS activity (data not shown). This indicated that (i) the defects were not just restricted to leaf cells and (ii) the possible existence of cell-type dependent differences in restriction of TEV infectivity of B149.

Further, the fact that the foci are not restricted when in contact with the midrib (junction of two cell types–Figure 3F) and at the edges of leaves (identical phenotype to that mentioned above when in contact with midrib–data not shown); i.e., when not surrounded by leaf cells in one direction, indicated the existence of a non–cell autonomous mechanism operating to restrict TEV in B149. Indeed, foci in other locations of leaves tend to shrink at later time points (as indicated by reduction in size of GUS foci) while these foci at the edges of leaves or in contact with the midrib tend to slightly increase in size. The shape and location seem to suggest their spread (though less then wild type) seem to be along the edges of the sides surrounded by midrib cells or none at all. This fact suggests that the restriction is probably mediated by a multi-directional mechanism with the infected cell as foci i.e., involving cell layers in all sides and the absence of such a signal even in one direction leads to partial breakdown in restriction of infection foci (more in Discussion section).

## Discussion

RNA viruses constitute an important class of pathogens. In this study a positive strand RNA virus, TEV has been used to isolate an Arabidopsis mutant impaired in susceptibility. The mutant, B149, is impaired in susceptibility to TEV, but not to the related potyvirus TuMV or the carmovirus TCV. Analyses of the mutant reveal some mechanistic aspects of viral restriction viz., a non-cell autonomous signal and a genetic interaction operative in the restriction of viral infection. The monogenic recessive locus conferring suppressed leaf infectivity and cell-to-cell movement in B149 is allelic to two mutant alleles of *lsp1* (*lsp1-1* and *lsp 1-2*) obtained by a positive selection scheme based on the loss of phenotype after infection with TuMV. The *lsp1-1* mutant phenotypes (impairment of infectivity to both TEV and TuMV, as opposed to specific impairment to TEV but not to TuMV as in B149) are conferred by a mutation resulting in premature stop codons in the eukaryotic m7G Cap binding translation initiation factor eiF(iso)4E [40]. The mutation in eiF(iso)4E is shown to be causal for *lsp1-1* phenotype by complementation by the gene from wildtype.

B149 harbors a mutation in the splice acceptor site of the third intron of *eiF(iso)4E*, the gene affected in the *lsp1* mutants impaired in susceptibility to TEV and TuMV. But, segregation studies using molecular markers flanking the locus conferring the *lsp1-1* phenotype indicates that the monogenic recessive locus conferring the B149 phenotype does not map to the *lsp1*. In addition, the leaf infectivity phenotype analyzed here is not allelic to a third *lsp1* allele (*lsp1-3*). One possibility to account for these results is a genetic interaction creating a haplo-insufficient situation when B149 is paired with certain alleles of *lsp1*. Some possibilities for such an interaction are (i) a molecular interaction between the components affected in the B149 mutant and eiF(iso)4E–i.e., *lsp1*, (ii) the locus conferring wild-type or mutant phenotype in B149 acting to enforce some epigenetic or RNA interference/silencing based constraint overriding the dominant nature of the requirement eiF(iso)4E in some alleles, or (iii) a novel mechanism that remains to be uncovered.

It is interesting to note that *lsp1-3*, despite being reported as having the same mutation in eiF(iso)4E as *lsp1-2*, did not complement B149 in allelic complementation tests, whereas the other two *lsp1* alleles tested did. Further, *lsp1-3* is not affected in susceptibility to TEV (Table 2, this work) whereas *lsp1-1* and *lsp1-2* are [40]. Lellis et. al., do not report on the susceptibility of *lsp1-3* to TEV. It is possible that the mutation in an upstream intron of *lsp1-3* in addition to harboring the same mutation as *lsp1-2* in some way accounts for the difference in the ability of B149 to complement the TEV infection phenotype. The intriguing aspect that *lsp1-3* is not impaired in susceptibility to TEV as with another mutant C13-3 belonging to a different complementation group identified in that study (Table 2) also needs to be highlighted here.

The restriction of TEV foci on leaf edges and differences in spread on different leaf cell types in B149 suggest dysregulation of a non-cell autonomous propagative regulatory mechanism (converging to or moving away from the infection site) affecting TEV susceptibility. Two lines of evidence supports this hypothesis, viz., (i) the foci are not succinct when in contact with the midrib or on the midrib, and (ii) similar expansion of infection foci occur when at the edge of leaves. This would suggest that this non-cell autonomous mechanism travels to or from several cell layers to affect TEV infection at the site of infection as well as expansion to adjacent cells. The absence of this regulatory signal in even one direction leads to increased spread of foci. Increased expansion of foci when in contact with or on the midrib suggests that this regulatory signal either cannot propagate well across cell types or is of differing strength in different cell types. Possible mechanisms include a prime-ahead strategy (i.e., making the cells susceptible ahead of spread in and around the site of infection) or conversely breakdown of a preexisting cellular mechanism/machinery that affect signal(s) that traverse towards infected cell(s) that now serves to restrict the multiplication and spread of TEV in B149, both possibilities not being mutually exclusive. Examples of such propagative signals in plants related to pathogen spread are adaptive spread of silencing in response to viral infection and systemic acquired resistance to broad spectrum of pathogens upon infection with a pathogen causing HR at a primary site. The propagative nature of the RNA mediated gene silencing mechanism is evident in plants and other organisms [47]–[50]. None of these studies have focused on multidirectional availability of cell layers capable of sending or receiving such a signal. In plants a variety of macromolecules (proteins, RNA etc.) are also shown to be transported short and long distances through plasmodesmata and phloem (e.g., reviewed in [51]). Other well studied signaling mechanisms involving multicellular signal perception are morphogen gradients in development in many organisms and recently renewed efforts in signals involved in maintaining stem cell niches (some examples reviewed in [52]–[56]).

In conclusion, an Arabidopsis mutant B149 that is impaired in multiple aspects of susceptibility to TEV affecting a propagating molecular signal dependent on the presence of cells and appropriate cell types in all directions surrounding the infected cell has been identified and characterized. The mutant displays a genetic interaction leading to haploinsufficiency with some, but not all, alleles of the previously reported Arabidopsis mutant *lsp1* impaired in susceptibility to TuMV and TEV. Understanding the molecular signal and the genetic mechanism operative in B149 should have impact beyond the study of susceptibility of TEV, viz., other viral host interactions, propagative control mechanisms and possibly novel genetic mechanisms that constitute cellular machinery.

## Materials and Methods

### Virus preparation and plant inoculation

TEV-P450 and TEV-GUS were propagated in *Nicotiana tabacum cv Xanthi-nc*, and inoculum prepared as described earlier. The TEV-P450 and TEV-GUS inoculum were diluted in 20 mM Tris.Cl (pH 8.0) with 10 g/l carborundum, and inoculated at 75 psi using the airbrush inoculation technique described earlier [39]. For mutant screens, Arabidopsis plants were inoculated when they are about three weeks old. For phenotypic characterizations plant age varied between three and four weeks at the time of inoculation. TuMV and TCV were propagated in *Nicotiana benthamiana*. In case of TuMV inoculum was prepared from *Nicotiana benthamiana* leaves 6 dpi with TuMV, by grinding infected leaves in 20 volumes 20 mM Tris.Cl (pH 8.0), and inoculated with airbrush with 10 g/l carborundum. TCV was partially purified by grinding infected leaf tissues in 10 volumes of NaOAc pH 5.2 and held on ice for 1 h. The suspension was centrifuged at 12,000 rpm for 15 minutes. One fourth volume of 40% PEG 8000 with 1 M NaCl was added to the supernatant and maintained in ice overnight. After centrifugation at 12, 000 rpm for 20 minutes, the viral pellet was resuspended in 10 mM NaOAc (pH 5.5) [40]. Arabidopsis plants dusted with carborundum were inoculated with TCV using cotton tipped applicators.

### Screen conditions and proherbicide application

Screen conditions were in principle similar to methods described earlier [39], with modifications described below. About three week old C24 plants mutagenized with EMS (M2 generation) were inoculated with TEV-P450 twice, with two day interval. Inflorescence were removed 10 and 12 days later and the plants were sprayed with R7402 at 100 ga/acre. R7402 (from DuPont, kind gift of Dr. Dan O'Keefe) was prepared as 50 mg/L stock in 10 mM KOH and diluted for use in deionized water with 100 ul/L Silwet L-77 (Vac-In Stuff, Lehle Seeds). 900–1200 µg/L R7402 was used for different screens. A total of 33,000 plants were screened in five batches. For small scale analyses as in Figure1, plants were hand sprayed with 330 µg/L R7402 at 10 and 12 dpi with TEV-P450, after removing inflorescence.

### Secondary screening, DNA extraction and preliminary mapping and sequencing and data analysis

DNA was prepared from inflorescence tissue and used for mapping using two markers already described [40]. M3 generation plants were checked for infectivity of TEV-GUS to eliminate escapes in the different steps of screen and to eliminate mutants in the herbicide resistance pathway. Leaves were stained for GUS activity using X-gluc [57], in some cases the stained leaves were cleared off chlorophyll (bleached) with 75% ethanol. For systemic/long distance movement inflorescence tissue at the end of dominant bolts were subject to quantitative GUS activity assay [19]. For determining the sequence of *eiF(iso)4E* from C24 and B149, products from eight independent PCR reactions were pooled, and sequenced.

## Supporting Information

Figure S1 Whole plant leaf infectivity assay in wildtype C24 and mutant B149 plants inoculated with TEV-GUS. Plants were inoculated identically with TEV-GUS. Panels A and C are wildtype C24 plants, panels B and D are B149 plants. Whole plants (viz., all rosette leaves) were stained for GUS activity 4 dpi to observe TEV infection foci.
(8.47 MB TIF)

Figure S2 Symptoms after inoculation of wildtype C24 and mutant B149 plants with TCV. Symptoms shown were recorded 9 dpi (Panels A–F) and 11 dpi (Panels G–J). Uninoculated C24 plants are shown in panels A and H, and the mutant B149 in panels B and G. Other panels show symptomatic (yellowing and maceration) plants after inoculation TCV at 9 dpi (C24- panels D and F; B149- panels C and E) or 11 dpi (C24- panel J and B149 panel I). By 16 dpi plants of both genotypes were dead (not shown). These plants were grown in suboptimal conditions to contain TCV. Under these conditions the size difference between B149 and C24 was more pronounced. As mentioned in the text, this trait could be segregated away from the impaired TEV infectivity phenotype.
(9.02 MB TIF)
