# Supplementary figures and images for: A Multidirectional Non-Cell Autonomous Control and a Genetic Interaction Restricting Tobacco Etch Virus Susceptibility in Arabidopsis
Source: PLoS One. 2007 Oct 3;2(10):e985. doi: 10.1371/journal.pone.0000985 (PMC1991623; doi:10.1371/journal.pone.0000985)

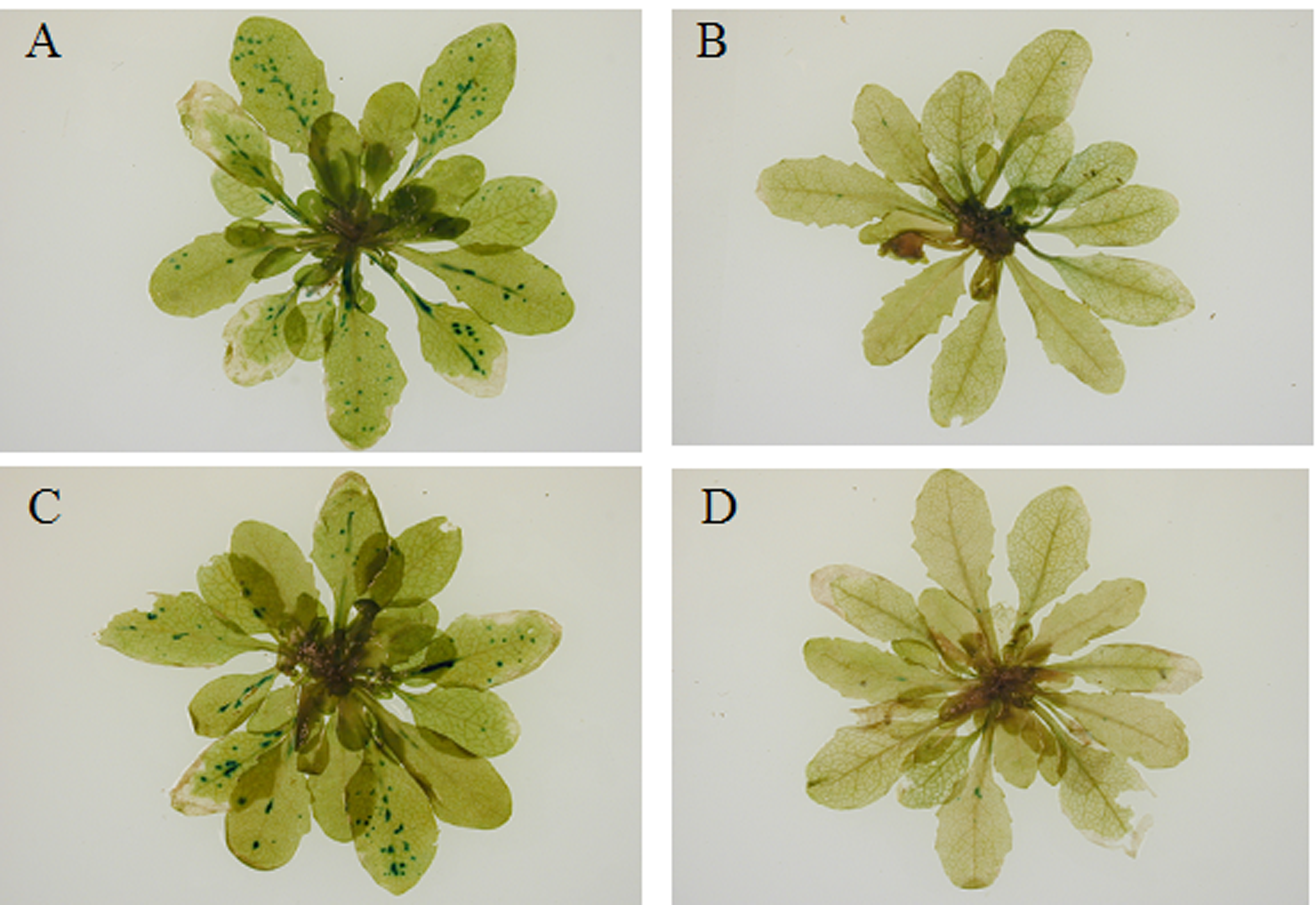

Supplement: Figure S1 — Whole plant leaf infectivity assay in wildtype C24 and mutant B149 plants inoculated with TEV-GUS. Plants were inoculated identically with TEV-GUS. Panels A and C are wildtype C24 plants, panels B and D are B149 plants. Whole plants (viz., all rosette leaves) were stained for GUS activity 4 dpi to observe TEV infection foci. (8.47 MB TIF) [file pone.0000985.s001.tif]

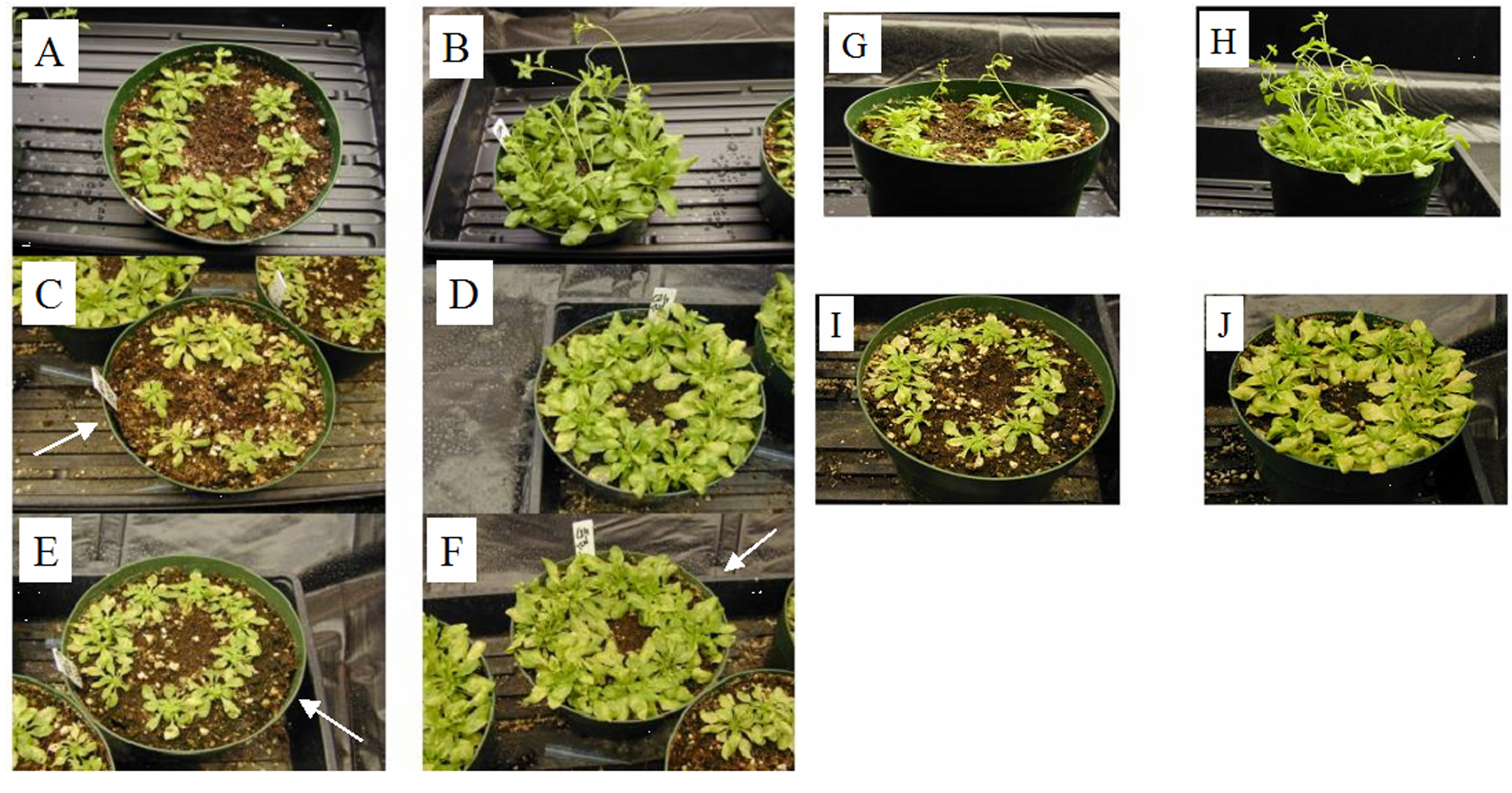

Supplement: Figure S2 — Symptoms after inoculation of wildtype C24 and mutant B149 plants with TCV. Symptoms shown were recorded 9 dpi (Panels A–F) and 11 dpi (Panels G–J). Uninoculated C24 plants are shown in panels A and H, and the mutant B149 in panels B and G. Other panels show symptomatic (yellowing and maceration) plants after inoculation TCV at 9 dpi (C24- panels D and F; B149- panels C and E) or 11 dpi (C24- panel J and B149 panel I). By 16 dpi plants of both genotypes were dead (not shown). These plants were grown in suboptimal conditions to contain TCV. Under these conditions the size difference between B149 and C24 was more pronounced. As mentioned in the text, this trait could be segregated away from the impaired TEV infectivity phenotype. (9.02 MB TIF) [file pone.0000985.s002.tif]
